# Epidemic Erysipelas, Called “Black Tongue”

**Published:** 1844-06

**Authors:** 


### ILLINOIS

### MEDICAL & SURGICAL JOURNAL.

VOL. I. JUNE, 1844. NO. 3.

### EPIDEMIC ERYSIPELAS, CALLED "BLACK TONGUE."

*Continued from page 22, last No.*

The *prognosis* of the disease, according to Drs. Hall and Dexter, "was governed, as in other disorders, by the age, sex, and condition of the patient, the organs and textures affected." When the skin was the seat of the disease, without affection of the subcutaneous cellular membrane, the cases were mild: the strongest constitutions would, however, give way before the extensive burrowing of the suppurative process, and the sloughing which usually followed the affection of the latter tissue. "The most fatal results, for the most part, were to be anticipated in the affection of the internal organs, particularly the bowels and uterus, and when the epidemic might be said to be at its height, not one in seven escaped, who had disease of the last mentioned organ."

The connection of erysipelas with *puerperal peritonitis* is clearly proved by the testimony of all who have observed this epidemic. Cases are related also, showing that the contagion is communicated to females who, during labor or confinement, are attended by physicians having under treatment patients affected with erysipelas. Whether communicated by the hand or clothes of the physician is not determined. The practical caution however is readily deducible, either to refuse to attend women in the puerperal state, during attendance upon cases of erysipelas; or at least, previously to change every article of apparel and perform personal ablutions to the fullest extent.

The fatality attending this epidemic, among females, renders every precaution of this kind imperative. "In the county of Caledonia, Vt., thirty cases of puerperal peritonitis occurred, *only one of which* recovered! And in Bath, N. H., containing a population of 1500 or 1600, twenty mothers died from puerperal peritonitis, and about forty with erysipelas." (Hall & Dexter, loc. cit.)

Post mortem examinations have been few, physicians having been deterred therefrom, by the death of several, consequent upon wounds received during dissection, and the narrow escape of others who contracted the disease from the same cause. In the few examinations reported, to which we have had access, death had occurred from inflammation of internal organs. In three cases in which the disease was seated in the abdominal cavity, the peritoneum was much injected and dark colored, in one instance with patches resembling gangrene. Serous effusions of a foul character had occurred to some extent in two of the cases, and in the third in which the patient died of puerperal peritonitis, "two quarts and a pint of pure pus were removed from the abdominal cavity, and every other organ exhibited signs of intense inflammation, excepting the uterus," which was free from disease and merely contained small coagula.

Cases have occurred in Chicago and its neighborhood, during a few months past, showing a tendency in inflammatory diseases, to assume a form similar to this epidemic. One case occurring in town, in the month of March, was in almost every symptom identical with cases mentioned by Drs. Hall and Dexter. In a family resident eight miles N. W. of the city, the disease assumed three different appearances noticed as simultaneously occurring wherever the epidemic has prevailed. The father was attacked with erysipelas of the face and neck, the oldest child, aged 12, had an abscess in the axilla, which upon being opened discharged a large amount of pus, a younger child had a similar tumor which was dissipated without discharging externally, and the mother who was confined during the illness of her husband was seized with puerperal mania in its most malignant form.

In the *treatment* of this disease much discrepancy is found in the accounts of the different observers, principally however with regard to the propriety of bleeding in the early stages. Dr. Jewett of Caledonia, Vt., strenuously condemns bleeding, and even doubts the propriety of the early administration of emetics and cathartics. Stimulating diaphoretics, counter-irritants, with anodynes when there was much restlessness, form the principal part of his treatment. "In many cases," he remarks, "and especially those where external erysipelas prevailed, and in some others an early resort was had to sulph. quinine, carbonate ammonia, camphor, and in some few cases, wine or the more active alcoholic stimulants were used with benefit." Drs. Hall and Dexter recommend bleeding and other antiphlogistic agents as strongly as others condemn the same practice. We quote their opinions.

"We have said that the disorder proved less fatal when confined to the mucous surfaces, than when transferred to any of the other structures. If such be the case, our efforts should be exerted to relieve the congestion of these surfaces, and to restore the circulation to its natural state as soon as possible. Can this be done by stimulating diaphoretics alone, without the aid of other depletion?

"We doubt not this has been done, when the affection of this membrane has been merely local, without any constitutional participation. But when in connection with this state of the membrane of the throat, we have extreme heat of skin; full, bounding, and frequent pulse; violent pain in the head, back, and limbs, and extreme thirst, there can be no doubt what course we should pursue. Bleeding, prompt and efficient bleeding is in such a state the only remedy to be depended upon, and in our hands the only one which has succeeded.

"A delay of a few hours in such a condition of affairs is fatal. Full bleeding, reducing the action of the heart and arteries, followed by either an emetic or cathartic, has rarely failed in our hands of arresting the disease, when applied in season. And not in a few cases where there has been but little affection of this membrane, but much efflorescence upon the skin, has one bleeding arrested the disease, and the patient become convalescent in a few days.

"These are the important aids to be depended upon, under the condition of circumstances mentioned; not, however, to the exclusion of those remedial agents which serve to carry out still farther the intention of such treatment. After bleeding, either a cathartic or emetic, followed by the pulvis Doveri cum antimonialis, and the free admission of mucilaginous drinks, and in those cases where there is much biliary disturbance, ipecac. combined with calomel, rarely fails to accomplish a cure. These indications are to be immediately acted upon. Erysipelas, like typhus, has its inflammatory stage, as well as a stage of collapse, and our efforts should be directed to arresting the disease before the period of collapse ensues. The great object of equalizing the circulation and restoring the vital energies of the system, may have been effected by sudorifics alone. But should we depend in the onset of typhus on sage tea, Dover's powders, and profuse perspiration?"

In Indiana, the same subject was discussed. Dr. Sutton is of opinion that bleeding from a large orifice was of great benefit, from the shock which it produced upon the system. He says, "A large blood-letting *from a small orifice* seldom failed to produce injurious effects, neither did patients bear a second venesection well, particularly in the pneumonia." He further says upon this subject, "If there has been any remedy in the course of treatment, that has caused this disease to be less fatal in this neighborhood than it has in other parts of the country over which it passed, it has been the prompt exhibition of an emetic, after venesection, making a decided impression upon the disease at its very onset, without prostrating the system. After this, calomel, opium, and antimony, in combination, followed by gentle laxatives, antimonial solutions, blisters, mucilages, and a light diet, was the principal course of treatment." Dr. S. remarks, upon the use of mercury, that a few doses generally filled the indication, and that great caution was necessary to avoid ptyalism, which he believes to have been almost invariably attended by injurious consequences.

These diverse opinions can we think result only from the fact, that in some sections the disease was more essentially typhoid in its character than in others. In the stages of collapse, all the writers upon the subject recommend, as a matter of course, resort to the diffusible stimuli, tonics, and nourishment.

The topical applications recommended, when the skin is the seat of the erysipelatous inflammation, are various, and appear to have met with varied success, Solutions of muriate of ammonia, nitrate of silver, sulphate of iron, sulphate of copper, iodine, blisters, acupuncturation all produced in some cases good effects, and in others completely failed to check the progress of the disease and prevent its ranging the whole surface. No treatment seems to have been effective in preventing suppuration or checking its advances when the cellular membrane was the seat of disease. In such instances all that remained for the physician to do was to evacuate the pus by incisions, remove the sloughs, and support the system.

